# Reviving Nitrogen-Vacancy Centers in Diamond via Local Surface Modification

**DOI:** 10.1021/acs.nanolett.5c03633

**Published:** 2025-08-23

**Authors:** Sergei Trofimov, Merve Aytac, Miriam Mendoza Delgado, Tommaso Pregnolato, Doguscan Ahiboz, Anna Makarova, Maxim Krivenkov, Oliver Rader, Tim Schröder, Cyril Popov, Boris Naydenov

**Affiliations:** † Berlin Joint EPR Laboratory and Department Spins in Energy Conversion and Quantum Information Science (ASPIN), 28340 Helmholtz-Zentrum Berlin für Materialien und Energie, Hahn-Meitner-Platz 1, 14109 Berlin, Germany; ‡ Institute of Nanostructure Technologies and Analytics (INA), Center for Interdisciplinary Nanostructure Science and Technology (CINSaT), 9178 University of Kassel, Heinrich-Plett-Str. 40, 34132 Kassel, Germany; ¶ Department of Physics, 9373 Humboldt-Universität zu Berlin, Newtonstraße 15, 12489 Berlin, Germany; § 28347 Ferdinand-Braun-Institut (FBH), Gustav-Kirchhoff-Straße 4, 12489 Berlin, Germany; ∥ Spin and Topology in Quantum Materials, Helmholtz-Zentrum Berlin für Materialien und Energie, Albert-Einstein-Straße 15, 12489 Berlin, Germany

**Keywords:** NV center, charge state, conductive AFM, surface oxidation

## Abstract

Surface termination of semiconductors is important for their applications in electronics because it governs electrical properties at interfaces. For quantum sensors and qubits based on spins in solids, this is crucial when they are located a few nanometers below the crystal surface. In the case of diamond, oxygen termination is preferential for quantum sensing with nitrogen-vacancy (NV) centers. Here, we present local surface modification of a nonconductive diamond surface utilizing conductive atomic force microscopy under laser illumination. By applying this method, we demonstrate not only a removal of fluorescence background but also control over the charge state of single NV centers. The latter show an improvement of the optically detected magnetic resonance contrast from 1% up to 29% after the treatment. We assume that local surface oxidation is happening on the diamond, which has already been demonstrated for conducting hydrogenated surfaces, but its implementation to nonconductive surfaces remains challenging.

## Graphical abstract

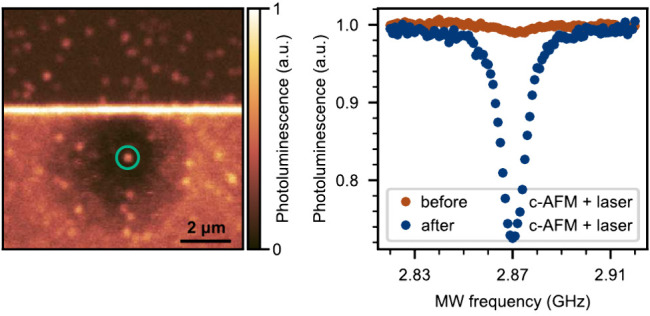

Diamond is a wide-band-gap (5.47 eV) material that has been considered for applications in electronics,^1^ photonics,^2^ quantum sensing and computing,^3,4^ electrochemistry,^5^ and medicine.^6^ These applications typically demand a certain surface termination, which defines the electronic properties of the diamond surface. In particular, the surface termination can be used to control the charge state of sensing qubits, which is routinely applied to shallow nitrogen-vacancy (NV) and silicon-vacancy (SiV) centers in diamond.^7,8^

Oxygen termination is preferential for quantum sensing because it was shown to preserve the negative charge state of NV centers (NV^–^),^9^ while hydrogen termination results in the Fermi level positioned below the valence-band maximum, which leads to neutral (NV^0^) and even positive (NV^+^) charge states of shallow NVs.^10^ The presence of C=C bonds (sp^2^ hybridization) on the diamond surface was also demonstrated to affect the NV centers, bringing them to the NV^0^ state.^11^ Less common types of terminating agents on the diamond surface include nitrogen,^12^ fluorine,^13^ and silicon.^14^

While macroscale surface termination is typically produced via boiling in acids or plasma etching/treatment,^15–17^ local surface modification can be achieved by utilizing scanning probe microscopy techniques.^18^ For diamond, the possibility of changing a hydrogen surface termination to an oxygen one was demonstrated using conductive atomic force microscopy (c-AFM).^19,20^ This technique is based on electric-field-assisted local electrochemical oxidation in the vicinity of the AFM cantilever tip at ambient conditions. The required surface conductivity in the case of diamond is provided by hydrogen termination. However, the local oxidation of surfaces with terminating groups that do not guarantee a high surface conductivity is challenging.

We report here the local surface oxidation of a nonconductive diamond surface using the c-AFM technique under green (λ = 520 nm) laser illumination. This allows not only removal of the fluorescence background at the submicroscale but also control of the charge state of single NV centers.

The sample used in this work is a (001)-oriented 4 × 4 × 0.05 mm IIa diamond plate grown by using a chemical vapor deposition method.

Part of the sample was subjected to nitrogen (^15^N^+^) implantation with an energy of 30 keV (corresponding to ≈45 nm penetration depth) and a dose of 3 × 10^9^ ions/cm^2^, while the other part was covered during the implantation. To create NV centers, the sample was annealed in a vacuum at 1000 °C for 2 h. After the annealing step, the sample was boiled in a 1:1 mixture of HNO_3_ and H_2_SO_4_ to remove the graphitized surface. Before the lithography step, the sample was cleaned in acetone and isopropyl alcohol.

Afterwards, the presence of NV centers was confirmed with a confocal photoluminescence (PL) mapping (Figure 1a,b).

**Figure 1:**
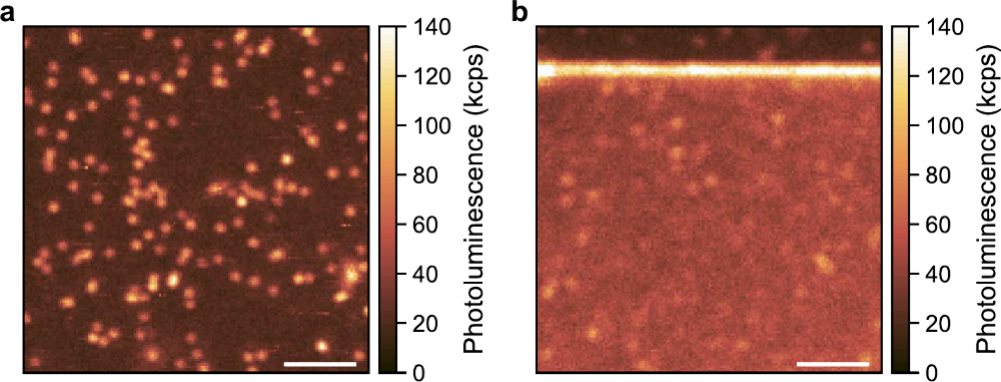
(a) PL map of the sample showing NV centers before metal deposition. (b) PL map of the sample after metal deposition. The biased electrode is visible in the top of the scan. The laser power in both experiments was 500 μW. The scale bars are 2 μm.

Finally, gold electrodes for the application of microwave (MW) signals and bias voltages were deposited on the sample surface using electron-beam lithography. A photograph of the sample with metal electrodes is shown in Figure 2a. A more detailed description of the sample preparation procedures can be found in the Supporting Information (SI).

**Figure 2:**
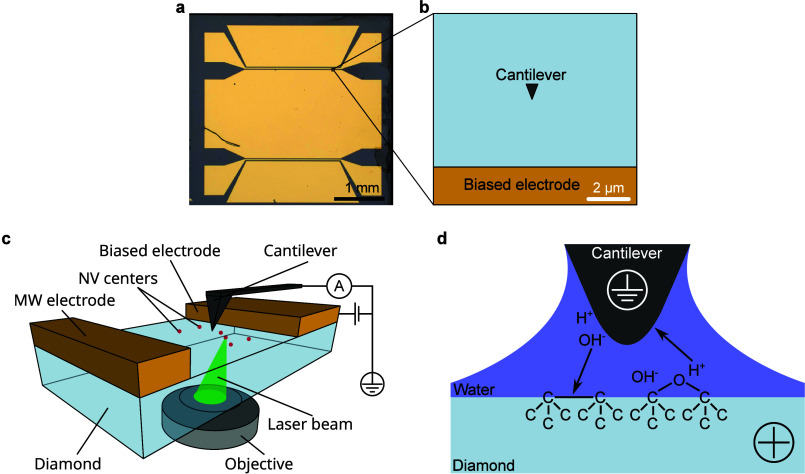
(a) Diamond plate with metal electrodes used in the experiments. (b) Schematic (2D, view from the top) of the typical experimental configuration (e.g., for the one in Figure 3). (c) 3D schematic of the c-AFM experiments under laser illumination. (d) Schematic of the processes occurring between the cantilever and sample under application of the positive bias to the sample.

The PL mapping conducted after the fabrication of surface electrodes reveals an increased background luminescence of the sample (Figure 1b), suggesting a change in the surface properties. As shown below, the NV centers are found to predominantly be in the neutral charge state, which points to a change in the electronic properties of the surface after the electrode manufacturing steps.

To determine if the additional fluorescence could be removed, the sample was cleaned in acetone using a sonicator, but the background PL still remained after this treatment. More harsh cleaning procedures could not be applied without destroying the gold MW structure. Interestingly, by performing conductive AFM experiments under laser illumination, we can change the surface properties. These measurements were conducted on a combined confocal microscopy–AFM setup consisting of an AFM and a home-built confocal microscope (see the SI) with a laser scanning technique,^21^ where the laser focal point was scanned around a cantilever that was fixed in place and in contact with the sample surface (Figure 2b). Here, we refer to contact as the AFM tip being in contact mode. During scanning, both the recorded PL and the electric current through the cantilever are correlated with the lateral position of the laser to obtain 2D PL and photocurrent maps simultaneously. A schematic of the experiment is shown in Figure 2c. In the following, we treat this experimental configuration as a metal–semiconductor–metal (MSM) structure,^22^ with the Schottky-type contact at the cantilever side and an ohmic contact at the surface electrode side. On the basis of our findings, we attribute the origin of the photocurrent to a photoexcitation of valence-band electrons to states at the metal–diamond interface (cantilever or bias electrode in Figure 3b at −10 or +10 V, respectively) and to surface states.^23,24^ This results in the generation of holes in the diamond valence band, which act as the main charge carriers moving in the applied electric potential.

**Figure 3:**
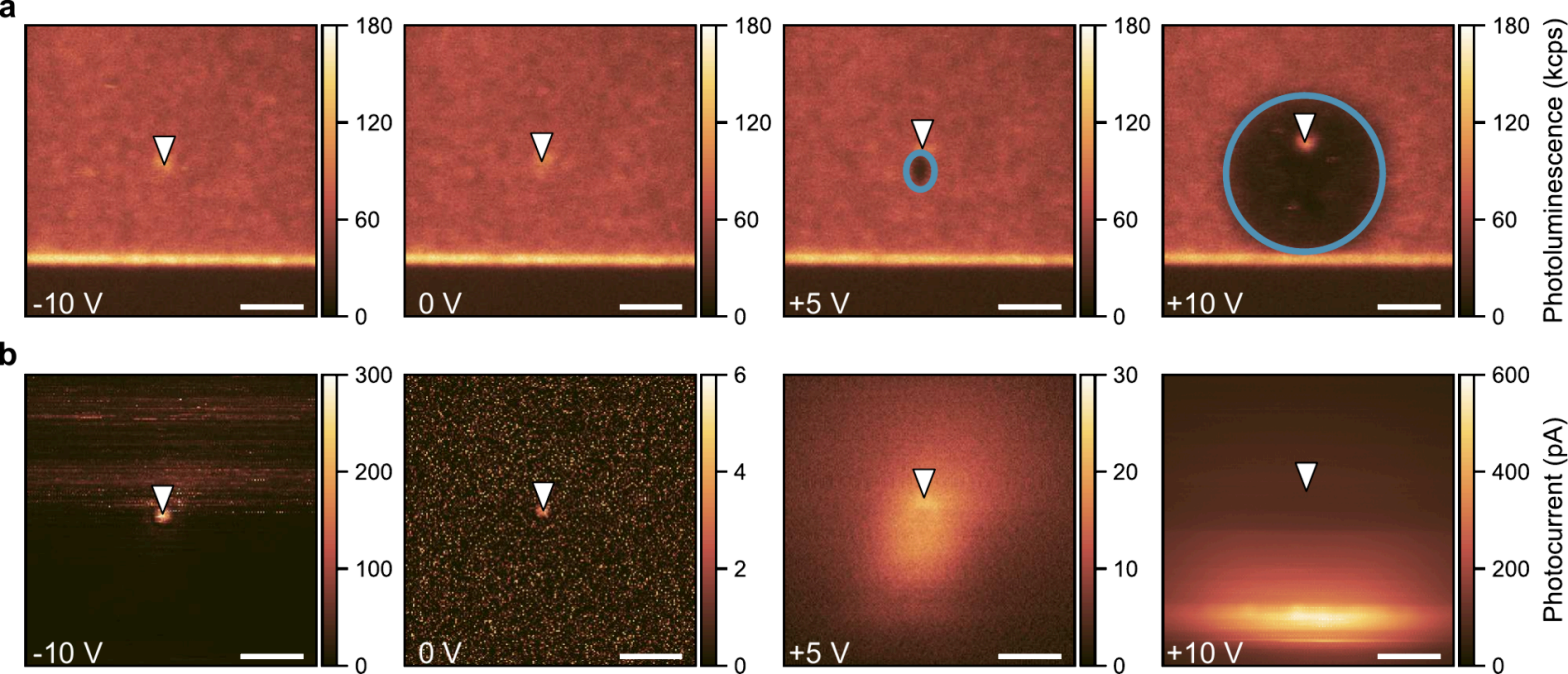
(a) PL maps of the sample surface in the nonimplanted region obtained at different bias voltages during c-AFM experiments. The biased electrode is located at the bottom of each scan (darker region). The cantilever, located in the center of each scan (indicated by a white triangle), is grounded. The PL maps at +5 and +10 V demonstrate the PL-bleached area (circled in blue) between the cantilever and the biased electrode (see also Figure 2b). (b) Photocurrent maps (absolute photocurrent) of the sample surface obtained at different bias voltages simultaneously with part a, as described in the main text. The laser power in all experiments was 500 μW. The scale bars are 2 μm.

Figure 3 shows the PL from the diamond surface as a function of the bias applied to the bias electrode. The data were measured in an area of the sample that was not subjected to ion implantation to exclude its possible influence on the results. From the data, we can conclude that the application of a negative bias (up to −10 V) to the electrode does not lead to changes in the surface PL. As can be seen in Figure 3b, in these conditions, the maximum photocurrent of about 300 pA is induced when the laser is focused on the grounded cantilever tip (left panel). This agrees with the assumption that the photogenerated holes are the main charge carriers (Figure S1) because they are efficiently generated at the metal–diamond interface with a higher potential^23^ and move to the lower potential electrode.

At 0 V, a low photocurrent on the order of 5 pA is induced when the laser is illuminating the cantilever, which can be explained by the presence of built-in voltage (Schottky barrier) at the tip–semiconductor interface.

A positive bias of +5 V results in a photocurrent on the order of 30 pA when the laser is focused between the cantilever and the biased electrode. This behavior can be explained by the fact that, at intermediate voltages lower than the Schottky barrier, the holes generated at the positively biased contact cannot reach the cantilever due to the bending. The current in this case originates only from populating the diamond surface states. Because the difference between the Schottky barriers can hardly be on the order of 5 V, it is also assumed that there is a series resistance (e.g., contact resistance, the water layer between the cantilever and the sample surface) that reduces the potential bias applied to the MSM structure.

At the bias voltage of +10 V, the maximum photocurrent of approximately 600 pA is observed when the laser illuminates the edge of the metal structure. This agrees with the assumption of positive charge carriers (holes). The linear dependence of the photocurrent on the laser power at +10 V (Figure S3a) suggests that the observed effect is a one-photon process, which agrees with previous observations of the photogenerated hole current at metal–diamond interfaces.^23^

We performed numerical simulations of a model 1D MSM structure using the *AFORS-HET* ^25^ software, which confirms the above-explained mechanism (Figure S1).

As demonstrated in Figure 3a, the background PL is significantly quenched (by ≈3 times) around the cantilever tip under the application of a positive bias to the surface electrode. In Figure S3b, the area with the bleached PL is plotted as a function of the total charge flown through the cantilever during the scan *q* = ∑_*i*_ *I*_*i*_ *t*_*i*_, where *I*_*i*_ is the photocurrent in the *i*th pixel and *t*_*i*_ is the laser dwell time per pixel. The observed linear dependence suggests that the bleaching effect is triggered by trapping of the charge carriers (holes) by defect states on the surface. Due to a small cross-sectional area of the cantilever–diamond contact, the high current density in the vicinity of the cantilever leads to a visible PL quenching effect, which was not observed when the potential was applied directly between two surface electrodes (MW and bias electrodes in Figure 2c).

Kelvin probe force microscopy (KPFM) measurements of the diamond surface indicate a lower work function (by ≈100 mV) of the PL-bleached area compared to the unaltered surface (Figure S4). This could be caused either by the presence of locally trapped charges, by a different material adsorbed on the surface, or by a change of the diamond surface termination.^26^ Because the induced changes in PL cannot be reverted by a photocurrent with reversed polarity and are stable for several weeks in ambient conditions, we exclude the local charging of the diamond surface as a possible reason for the photocurrent-induced PL bleaching. A different material on the diamond surface could be residuals of the PMMA-based resist left after the electron-beam lithography process. However, these residues should be completely removed after the sample is sonicated in acetone. Therefore, we attribute the observed effects to a change in diamond surface termination. In an attempt to obtain more information about the changes happening on the surface, we performed X-ray photoelectron spectroscopy (XPS) on this sample and compared it with a reference sample, which underwent the same lithography procedure. Unfortunately, the area modified during c-AFM was too small to be able to detect an XPS signal from that region. Nevertheless, XPS spectra could be obtained from other sample areas, where the C 1s peak shows a slight difference compared to the reference (see the SI for more details), but more investigation is needed in order to resolve the chemical structure.

The most stable common diamond surface terminating agents, in order of increasing work function, are hydrogen, oxygen, and carbon sp^2^ hybridization.^26–28^ Because we do not detect an electric current in the absence of laser excitation, we exclude the presence of hydrogen termination. KPFM measurements (Figure S4c) and the results obtained with NV centers shown below suggest, that in our c-AFM experiments under laser illumination, the amount of carbon sp^2^ hybridization at the diamond surface reduces, while the percentage of oxygen termination increases. However, this could not be confirmed by XPS because no signal could be detected from the c-AFM-treated area.

The illustration of this mechanism is presented in Figure 2d. Because the experiments are conducted at ambient conditions (a temperature of 25 °C and a relative humidity of 40%), a water meniscus is formed between the cantilever tip and the surface.^29,30^ While we could not perform a complete contact-angle measurement due to the small size of the chemically modified area, a wetting (by a drop of water) of the diamond surface was clearly observed under a microscope.

The water serves as an electrolyte where anodic oxidation occurs at the sample–water interface:^31–33^

1 $$C_{2}+{\mathrm{OH}}^{-}\to C_{2}O+H^{+}+2e^{-}$$

while a counter reaction occurs at the water–cantilever interface:

2 $$H_{2}O+H^{+}+2e^{-}\to {\mathrm{OH}}^{-}+H_{2}$$

Here C_2_ represents the C–C surface bond (sp^2^ hybridization) and C_2_O stands for the C–O–C surface configuration, as shown in Figure 2d.

While in noncontact AFM experiments the size of the oxidized region is limited by the size of the water meniscus,^34^ in contact mode under the continuous application of a current, the oxyanions start to diffuse laterally due to space charge effects.^35^ This leads to an increase of the lateral size of the oxidized area, which in our case reaches up to 30 μm^2^ (Figure S3b).

Local modification of the surface termination can be beneficial for preserving certain charge states of color centers. We demonstrate this on NV centers created in the implanted region of the sample. Here, the application of the photocurrent reduces the background PL, while the NV center PL remains almost at the same level (Figure 4a,b).

**Figure 4:**
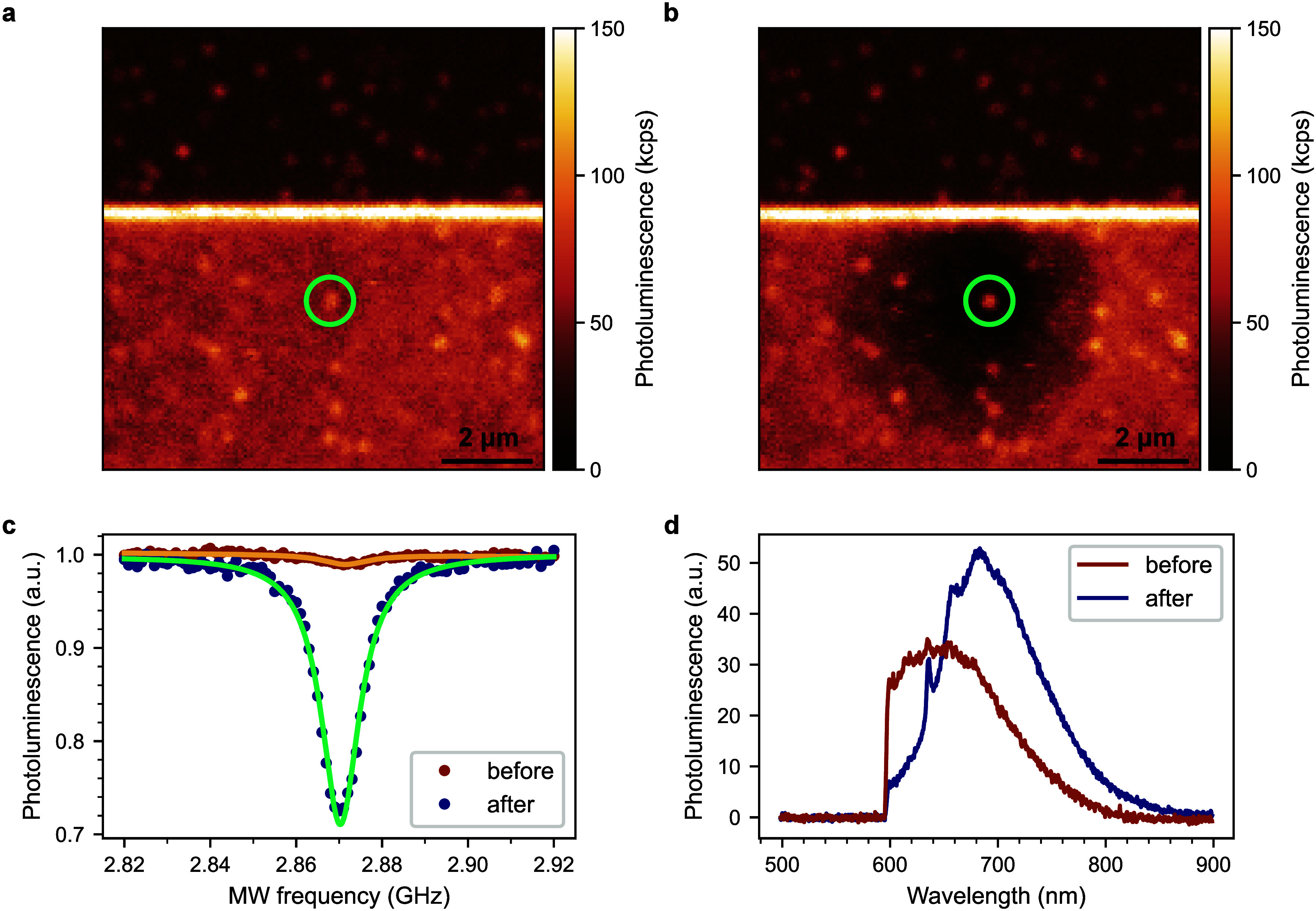
(a) PL map of the sample surface showing single NV centers before application of the photocurrent. (b) PL map of the same area after application of the photocurrent and retraction of the cantilever from the surface. During photocurrent application, the cantilever was placed 3 μm away from the surface electrode (located in the top) that was at +10 V potential. The light-green circle indicates the NV from which the ODMR and PL spectra shown below were obtained. (c) ODMR spectra on the single NV center before (brown points) and after (dark-blue points) photocurrent application. Each data set is fitted with a Lorentzian function (orange and green lines, correspondingly). The ODMR contrast increased from 1% up to 29% after application of a photocurrent. (d) PL spectra from the single NV center (background subtracted) before (brown) and after (dark blue) application of a photocurrent.

To probe the predominant charge state of the NV centers before and after the c-AFM experiments, we first used the optically detected magnetic resonance (ODMR) technique. In this method, the NV center PL is monitored during the application of MWs through the MW electrode as a function of their frequency. The negatively charged NV^–^ center exhibits a magnetic resonance at a frequency of 2.87 GHz (at zero magnetic field), which results in a decrease in PL.^36^ The NV^0^ center shows no magnetic resonance in such an experiment. As demonstrated in Figure 4c, the ODMR contrast of NV centers, defined as $C=\frac{\left|I_{\mathrm{res}}-I_{\mathrm{off}}\right|}{I_{\mathrm{off}}}$ , where *I*_res_ and *I*_off_ are the resonance and off-resonance NV PL, respectively, increased significantly from approximately 1% to 29% after the c-AFM surface treatment. This observation shows that the application of c-AFM under laser illumination influences the NV charge state likely caused by the local oxidation of the diamond surface.^11^

PL spectra illustrating changes in the PL of the NV center are shown in Figure 4d. The data demonstrate the transition of the NV center from the predominantly neutral charge state NV^0^ before application of the photocurrent to a predominantly negative charge state NV^–^ with the characteristic zero-phonon line at 637 nm after PL bleaching.^36^ This shows that we were able to “revive” the NV^–^ charge state by applying this technique, similarly to previously demonstrated shallow NV revival at ultrahigh-vacuum and low-temperature conditions due to water adsorption.^7^ The background PL shown in Figure S5a represents a wide spectrum with a maximum at a wavelength of 650 nm.

To analyze how improvement of the ODMR contrast depends on the NV position in the confocal scans, we measured ODMR on several NV centers before (*C*_i_) and after (*C*_f_) photocurrent application. The difference between the ODMR contrasts (*C*_f_ – *C*_i_) as a function of the distance from the NVs to the border between the bright and dark regions after photocurrent application is shown in Figure 5. The data demonstrate that all measured NV centers located inside the dark region (positive distances) show a significant improvement of the ODMR contrast (up to about 29%). The NV centers located at the border show a moderate increase in the ODMR contrast of about 15%, while for NVs further than 0.5 μm from the border (negative distances), the ODMR contrast improvement drops below 10%. The ODMR contrast values used to obtain this dependence are presented in Table S1.

**Figure 5:**
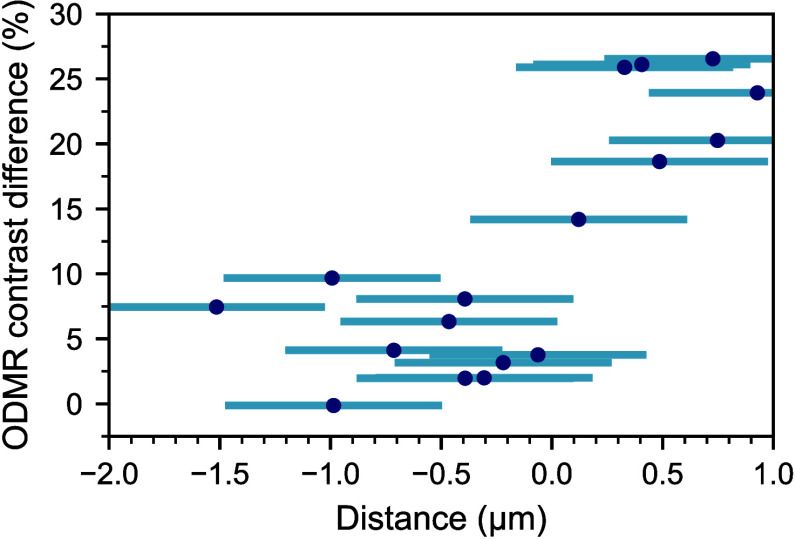
Dependence of the ODMR contrast difference *C*_f_ – *C*_i_ on the distance between NVs and the border of the bleached area. Positive (negative) distances correspond to NVs being inside (outside) the bleached region. The larger error bars are due to the poorly defined border between the two areas.

In conclusion, we demonstrated here the local oxidation of a diamond surface at submicron spatial dimensions using c-AFM during laser illumination. The observed effect opens a novel way for extending the existing local anodic oxidation technique to semiconductors exhibiting photoconductivity. The dependency of the area of the oxidized region on the duration time of the measured photocurrent allows one to control the resulting oxidized pattern by tuning the laser power, applied bias voltage, proximity to the biased electrode, and scanning rate.

The demonstrated benefits of the technique for NV centers highlight the potential applications of our method for the local charge state control. Given that the lateral size of the conductive cantilever tip is on the order of tens of nanometers, local surface modification could be useful in quantum sensing based on ensembles of NV centers for improving the spatial resolution of the sensor by creating an area with NV^–^ centers of a size that is less than the diffraction limit.

## Supplementary Material



## Notes

Materials, methods, photocurrent dependency on the laser power, bleached area dependency on charge, KPFM studies, PL spectra, and current–voltage characteristics (PDF)

## References

[1] Umezawa H. (2018). Recent advances
in diamond power semiconductor devices. Materials
Science in Semiconductor Processing.

[2] Mi S., Kiss M., Graziosi T., Quack N. (2020). Integrated photonic
devices in single crystal diamond. Journal of
Physics: Photonics.

[3] Segawa T. F., Igarashi R. (2023). Nanoscale quantum sensing with Nitrogen-Vacancy centers
in nanodiamonds – A magnetic resonance perspective. Prog. Nucl. Magn. Reson. Spectrosc..

[4] Pezzagna S., Meijer J. (2021). Quantum computer based
on color centers in diamond. Appl. Phys. Rev..

[5] Yang N., Yu S., Macpherson J. V., Einaga Y., Zhao H., Zhao G., Swain G. M., Jiang X. (2019). Conductive diamond:
synthesis, properties, and electrochemical applications. Chem. Soc. Rev..

[6] Mani N., Rifai A., Houshyar S., Booth M. A., Fox K. (2020). Diamond in
medical devices and sensors: An overview of diamond surfaces. Med. Devices Sens..

[7] Neethirajan J. N., Hache T., Paone D., Pinto D., Denisenko A., Stöhr R., Udvarhelyi P., Pershin A., Gali A., Wrachtrup J., Kern K., Singha A. (2023). Controlled Surface
Modification to Revive Shallow NV^–^ Centers. Nano Lett..

[8] Zhang Z.-H., Zuber J. A., Rodgers L. V., Gui X., Stevenson P., Li M., Batzer M., Grimau Puigibert M., Shields B. J., Edmonds A. M., Palmer N., Markham M. L., Cava R. J., Maletinsky P., De Leon N. P. (2023). Neutral Silicon
Vacancy Centers in Undoped Diamond
via Surface Control. Phys. Rev. Lett..

[9] Yamano H. (2017). Charge state stabilization
of shallow nitrogen vacancy centers in
diamond by oxygen surface modification. Jpn.
J. Appl. Phys..

[10] Hauf M. V., Grotz B., Naydenov B., Dankerl M., Pezzagna S., Meijer J., Jelezko F., Wrachtrup J., Stutzmann M., Reinhard F., Garrido J. A. (2011). Chemical control
of the charge state of nitrogen-vacancy centers in diamond. Phys. Rev. B.

[11] Stacey A., Dontschuk N., Chou J., Broadway D. A., Schenk A. K., Sear M. J., Tetienne J., Hoffman A., Prawer S., Pakes C. I., Tadich A., De Leon N. P., Gali A., Hollenberg L. C. L. (2019). Evidence for Primal sp^2^ Defects at the Diamond
Surface: Candidates for Electron Trapping and Noise Sources. Adv. Mater. Interfaces.

[12] Kawai S. (2019). Nitrogen-Terminated Diamond Surface for Nanoscale NMR by Shallow
Nitrogen-Vacancy Centers. J. Phys. Chem. C.

[13] Cui S., Hu E. L. (2013). Increased negatively
charged nitrogen-vacancy centers in fluorinated
diamond. Appl. Phys. Lett..

[14] Sear M. J., Schenk A. K., Tadich A., Stacey A., Pakes C. I. (2017). P-type
surface transfer doping of oxidised silicon terminated (100) diamond. Appl. Phys. Lett..

[15] Brown K. J., Chartier E., Sweet E. M., Hopper D. A., Bassett L. C. (2019). Cleaning
diamond surfaces using boiling acid treatment in a standard laboratory
chemical hood. Journal of Chemical Health &
Safety.

[16] Maki T., Shikama S., Komori M., Sakaguchi Y., Ken Sakuta K. S., Takeshi Kobayashi T.
K. (1992). Hydrogenating Effect
of Single-Crystal Diamond Surface. Jpn. J. Appl.
Phys..

[17] Li C., Zhang X., Oliveira E. F., Puthirath A. B., Neupane M. R., Weil J. D., Birdwell A. G., Ivanov T. G., Kong S., Gray T., Kannan H., Biswas A., Vajtai R., Galvao D. S., Ajayan P. M. (2021). Systematic
comparison
of various oxidation treatments on diamond surface. Carbon.

[18] Dagata J. A., Tseng W., Bennett J., Schneir J., Harary H. H. (1991). Nanolithography
on III-V semiconductor surfaces using a scanning tunneling microscope
operating in air. J. Appl. Phys..

[19] Tachiki M., Fukuda T., Sugata K., Seo H., Umezawa H., Kawarada H. (2000). Control of adsorbates and conduction
on CVD-grown diamond
surface, using scanning probe microscope. Appl.
Surf. Sci..

[20] Rezek B., Garrido J., Stutzmann M., Nebel C., Snidero E., Bergonzo P. (2002). Local Oxidation of
Hydrogenated Diamond Surfaces for
Device Fabrication. physica status solidi (a).

[21] Trofimov S., Naydenov B. (2025). Combined Confocal-Atomic-Force
Microscope Setup for
Quantum Sensing Applications with Sub-diffractional Spatial Resolution. Physica Status Solidi (a).

[22] Sze S., Coleman D., Loya A. (1971). Current transport in metal–semiconductor–metal
(MSM) structures. Solid-State Electron..

[23] Rieger M., Villafañe V., Todenhagen L. M., Matthies S., Appel S., Brandt M. S., Müller K., Finley J. J. (2024). Fast optoelectronic
charge state conversion of silicon vacancies in diamond. Sci. Adv..

[24] Chemin A., Levine I., Rusu M., Vaujour R., Knittel P., Reinke P., Hinrichs K., Unold T., Dittrich T., Petit T. (2023). Surface-Mediated Charge Transfer of Photogenerated Carriers in Diamond. Small Methods.

[25] Stangl, R. ; Leendertz, C. In Physics and Technology of Amorphous-Crystalline Heterostructure Silicon Solar Cells; Van Sark, W. G. J. H. M. , Korte, L. , Roca, F. , Eds.; Engineering Materials Series; Springer: Berlin, Heidelberg, 2012; pp 445–458.

[26] Rezek B., Nebel C. (2005). Kelvin force microscopy on diamond surfaces and devices. Diamond Relat. Mater..

[27] Tachiki M., Kaibara Y., Sumikawa Y., Shigeno M., Kanazawa H., Banno T., Soup Song K., Umezawa H., Kawarada H. (2005). Characterization
of locally modified diamond surface using Kelvin probe force microscope. Surf. Sci..

[28] Stehlik S., Petit T., Girard H. A., Arnault J.-C., Kromka A., Rezek B. (2013). Nanoparticles Assume Electrical Potential
According to Substrate,
Size, and Surface Termination. Langmuir.

[29] Hiroyuki
Sugimura H. S., Nobuyuki Nakagiri N.
N. (1995). Chemical Approach to
Nanofabrication: Modifications of Silicon Surfaces Patterned by Scanning
Probe Anodization. Jpn. J. Appl. Phys..

[30] Yuan Y., Lanza M. (2024). The Effect of Relative
Humidity in Conductive Atomic Force Microscopy. Adv. Mater..

[31] Sugimura H., Uchida T., Kitamura N., Masuhara H. (1994). Scanning Tunneling
Microscope Tip-Induced Anodization for Nanofabrication of Titanium. J. Phys. Chem..

[32] Garcia R., Martinez R. V., Martinez J. (2006). Nano-chemistry and scanning probe
nanolithographies. Chem. Soc. Rev..

[33] Tachiki M., Fukuda T., Sugata K., Seo H., Umezawa H., Kawarada H. (2000). Nanofabrication on Hydrogen-Terminated
Diamond Surfaces
by Atomic Force Microscope Probe-Induced Oxidation. Jpn. J. Appl. Phys..

[34] Garcia R., Calleja M., Rohrer H. (1999). Patterning of silicon surfaces with
noncontact atomic force microscopy: Field-induced formation of nanometer-size
water bridges. J. Appl. Phys..

[35] Dagata J. A., Perez-Murano F., Martin C., Kuramochi H., Yokoyama H. (2004). Current, charge, and
capacitance during scanning probe
oxidation of silicon. I. Maximum charge density and lateral diffusion. J. Appl. Phys..

[36] Gruber A., Dräbenstedt A., Tietz C., Fleury L., Wrachtrup J., Borczyskowski C. V. (1997). Scanning Confocal Optical Microscopy and Magnetic Resonance
on Single Defect Centers. Science.

