# Supplementary material for: Reviving Nitrogen-Vacancy Centers in Diamond via Local Surface Modification
Source: Nano Lett. 2025 Aug 23;25(35):13380–6. doi: 10.1021/acs.nanolett.5c03633 (PMC12412149; doi:10.1021/acs.nanolett.5c03633)
Supplement: Supplementary file 1 [file nl5c03633_si_001.pdf]

Supporting Information for

Reviving Nitrogen-Vacancy Centers in Diamond via Local Surface Modification

Sergei Trofimov[1,*], Merve Aytac[1], Miriam Mendoza Delgado[2], Tommaso Pregnolato[3,4], Doguscan Ahiboz[3,4], Anna Makarova[5], Maxim Krivenkov[5], Oliver Rader[5], Tim Schröder[3,4], Cyril Popov[2], and Boris Naydenov[1]

[1]*Berlin Joint EPR Laboratory and Department Spins in Energy Conversion and Quantum Information Science (ASPIN), Helmholtz-Zentrum Berlin für Materialien und Energie, Hahn-Meitner-Platz 1, 14109 Berlin, Germany*

[2]*Institute of Nanostructure Technologies and Analytics (INA), Center for Interdisciplinary Nanostructure Science and Technology (CINSaT), University of Kassel, Heinrich-Plett-Str. 40, 34132 Kassel, Germany*

[3]*Department of Physics, Humboldt-Universität zu Berlin, Newtonstraße 15, 12489 Berlin, Germany*

[4]*Ferdinand-Braun-Institut (FBH), Gustav-Kirchhoff-Straße 4, 12489 Berlin, Germany*

[5]*Spin and Topology in Quantum Materials, Helmholtz-Zentrum Berlin für Materialien und Energie, Hahn-Meitner-Platz 1, 14109 Berlin, Germany*

[*]sergei.trofimov@helmholtz-berlin.de

Contents

S1 Materials and Methods **2**
- S1.1 Diamond Sample . . . 2
- S1.2 Methods . . . 2

S2 Numerical simulations of the band structure **3**

S3 ODMR contrast spatial dependence **5**

S4 XPS Experiments **5**

S5 Supplementary figures **7**

S6 References **9**

S1 Materials and Methods

S1.1 Diamond Sample

The sample used in this work is a (001)-oriented 4 mm × 4 mm × 0.05 mm IIa diamond plate from AppliedDiamond grown using a chemical vapor deposition (CVD) method.

A part of the sample was subjected to a nitrogen $^{15}N^{+}$ implantation with an energy of 30 keV and a dose of 3×10^{9} ions/cm^2. The implantation of 30 keV was chosen to create NV centers deep enough (40–50 nm) to eliminate short-range dipole interaction with surface spins which limits the coherence time as reported previously [1,2]. At the same time the NVs should be close enough to the surface to be affected by the surface band bending [3,4]. After the implantation the sample was annealed in vacuum according to the following procedure: 1 hour ramping from room temperature (RT) to 500°C; 1 hour at 500°C; 1 hour ramping from 500°C to 1000°C; 2 hours at 1000°C; 1 hour ramping from 1000°C to RT.

After the annealing the sample was boiled in a 1:1 mixture of HNO_3 and H_2SO_4 to remove the graphitized surface. Before the lithography step the sample was cleaned in acetone and isopropanol.

Gold electrodes for the application of microwave (MW) signals and bias voltage were deposited on the sample surface via the following process. Two layers of PMMA (AR-P 617.06, Allresist) were sequentially spin-coated and baked at different temperatures: 230°C for the lower layer and 250°C for the upper layer. Once the desired pattern was transferred into the PMMA resist via electron beam lithography (EBL), metal deposition was carried out, starting with 10 nm of Ti as an adhesion layer, followed by 700 nm of Au in a Balzers BAK 600 Evaporation System. By immersion in TechniStrip D350, the underlying resist layers are dissolved, leaving metal only in the exposed areas.

S1.2 Methods

All measurements were performed on a combined confocal-AFM setup consisting of an AFM (NX12, Park Systems) and a home-built confocal microscope [5]. A green (λ = 520 nm) continuous wave (CW) laser diode (RLT520-80MGS, Roithner LaserTechnik) was used for all experiments. In magnetic resonance detection measurements, NV center spin transitions were excited by MWs generated by a CW microwave source (AWG7122C, Tektronix) and after amplification (ZVA-213-S+, Mini-Circuits) applied to the sample via the gold strip-line electrodes on the surface. Photoluminescence (PL) was detected with an avalanche photodiode (SPCM-AQRH-44, Excelitas), and PL spectra were measured using a spectrograph (IsoPlane 160, Teledyne). Qudi software suite [6] was used for controlling the optical part of the setup.

In the experiments on surface modification by application of a photocurrent (PC), an electrically grounded platinum cantilever (25Pt300B, Rocky Mountain Nanotechnology) was brought in contact with the diamond surface 2–3 µm away from the gold structures. A bias voltage up to ±20 V was applied to the gold electrode. The laser beam focused on the diamond surface was scanning around the cantilever position, inducing the photocurrent and the consequent surface modification. The photocurrent flowing through the cantilever was measured with a transimpedance amplifier (DLPCA-200, FEMTO). In KPFM experiments,

PtIr5-coated silicon cantilevers (EFM, NanoWorld) were utilized to visualize the differences in the surface potential after modification.

X-ray Photoelectron Spectroscopy (XPS) measurements were carried out at the GELEMPES instrument at the BESSY II electron storage ring operated by the Helmholtz-Zentrum Berlin für Materialien und Energie [7]. XPS spectra were acquired with a hemispherical Phoibos 150 electron energy analyzer (Specs GmbH) in normal emission geometry. XPS measurements were affected by positive charging due to the insulating nature of the diamond samples. To correct for this, the binding energy scale was calibrated by referencing the diamond sp^3 C 1s peak to 285 eV. All measurements were carried out at room temperature. The base pressure was 1×10^{-10} mbar. Peak fitting was performed on the C1s XPS data using CasaXPS analysis software.

S2 Numerical simulations of the band structure

To explain the photocurrent maps shown in Figure 3 of the main text, we conducted numerical simulations of the diamond energy band structure using the AFORS-HET software [8]. Our 1D model system consisted of a diamond layer with an acceptor concentration of $N_\mathrm{a} = 1 \times 10^{14}$ cm^{-3}, chosen to represent the low doping from acceptor surface states [9,10] and two Schottky contacts representing the surface electrode and the cantilever (metal-semiconductor-metal structure [11]). The work function of the cantilever contact was set to be 5.5 eV [12]. The surface electrode work function was set to 6.5 eV to simulate the ohmic type of the Ti-diamond interface [13]. The results of the simulations are shown in Figure S1. In order to excite electrons in the conduction band and holes in the valence band of diamond UV light should be applied [14, 15]. In our case however at zero bias voltage (Figure S1a), the holes excited by the laser from the surface trap states (E_t) at the cantilever position are able to reach the surface electrode due to the band bending, which leads to a small current, observable in Figure 3b at 0 V. At negative voltages applied to the surface electrode (Figure S1b), the bands continue to bend in the same direction, which results in the highest photocurrent located at the cantilever position (Figure 3b at -10 V). At small positive voltages (Figure S1c), the bands start to bend in the other direction. The holes excited from the surface states between the electrodes reach the cantilever, which leads to the observable photocurrent in Figure 3b at +5 V. However, the holes excited near the surface electrode location cannot reach the cantilever due to the local band bending. As the positive voltage increases (Figure S1d), the band bending becomes more preferable for the holes excited near the surface electrode to reach the cantilever (Figure 3b at +10 V). This simplified model allows to qualitatively explain the observed photocurrent maps, though to simulate the real experiment one would need to take into the account the geometry of the whole structure.

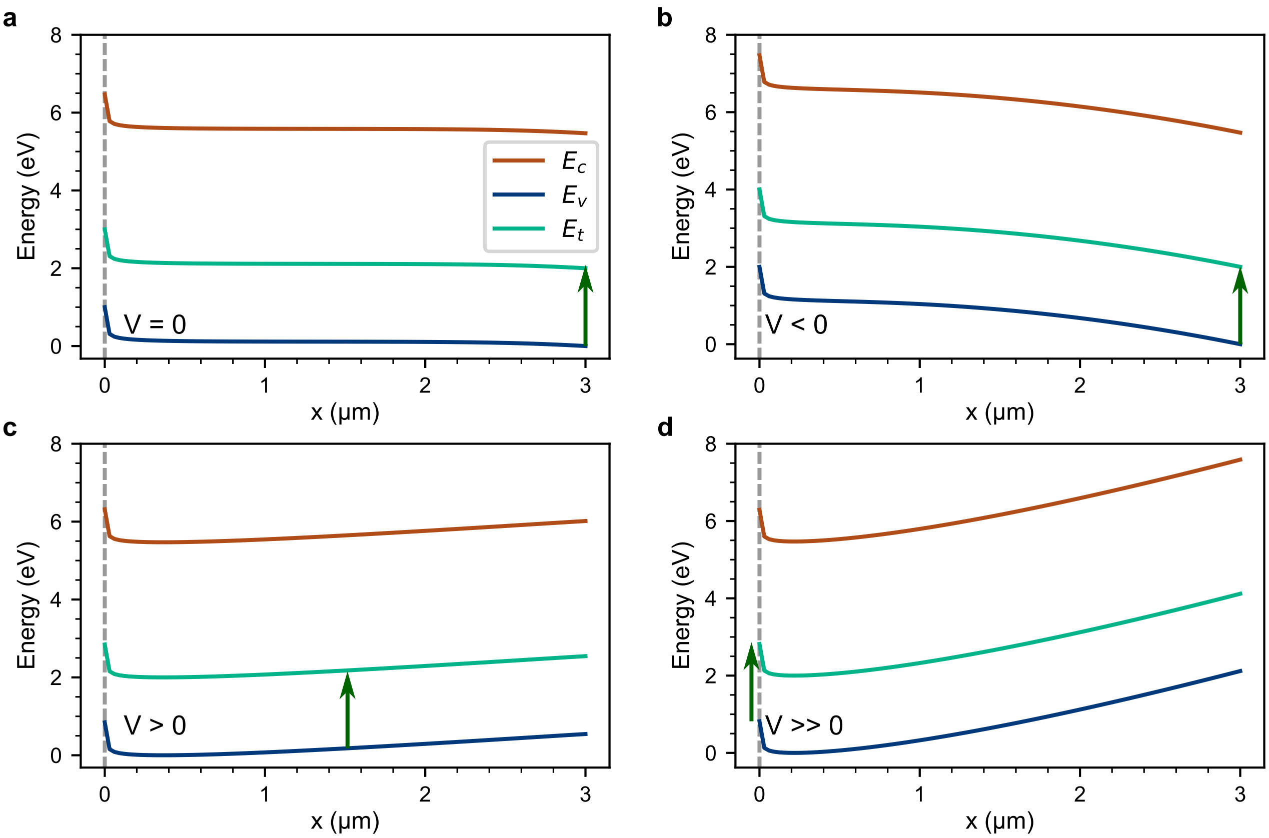

Figure S1: Band diagrams of a metal-semiconductor-metal diamond structure, simulated with AFORS-HET, showing the position of the diamond valence (E_v) and conduction (E_c) bands as well as the trap state (E_t) between the surface electrode (located at $x = 0$, gray line) and the cantilever (at $x = 3$) at different voltages applied to the surface contact. The green arrow indicates the laser excitation of holes to the diamond valence band. (a) Energy bands at zero voltage. (b) Energy bands at negative voltage. (c) Energy bands at small positive voltage. (d) Energy bands at high positive voltage.

S3 ODMR contrast spatial dependence

Spatial dependence of the ODMR contrast improvement was measured on 17 NV centers located inside and outside the bleached regions. The data is summarized in Table S1, where the distance from the NVs to the bleached region border is positive for NVs inside the bleached region and negative for the ones outside. The data is also plotted in Figure 5 of the main text.

Table S1: Change in the ODMR contrast.

	ODMR contrast, %		
NV	Before bleaching	After bleaching	Distance to the bleached region border, µm
1	0.88	7.21	−0.465
2	1.16	4.33	−0.220
3	0.81	26.92	0.406
4	0.81	27.36	0.727
5	0.76	21.03	0.748
6	0.84	15.03	0.121
7	0.39	8.46	−0.393
8	0.81	4.58	−0.063
9	1.35	27.24	0.328
10	1.85	11.53	−0.993
11	7.21	11.33	−0.714
12	0.79	19.43	0.486
13	0.66	2.63	−0.392
14	0.62	0.49	−0.986
15	2.25	9.70	−1.515
16	0.58	24.51	0.928
17	0.46	2.46	−0.306

S4 XPS Experiments

Since we cannot resolve the area of the sample treated by c-AFM tip in the synchrotron-radiation XPS setup, we performed comparative C 1s XPS on two diamond samples. The first one was used for the c-AFM experiments which showed high PL background and the second one was a diamond sample that went through the same lithography process, but showed later low PL background. C 1s core-level XPS spectra collected at photon energy of 645 eV together with results of the fitting analysis are shown in figure S2. The spectra exhibit rather similar structure: in addition to the major feature related to the sp^3-hybridized carbon we observe components both at lower and at higher binding energies (BEs). Features at higher BEs are related to different types of oxygen species on the surface. Peaks shifted by 1 eV, 2.1-2.4 eV and 3.2 eV from the major sp^3 one are related to C-O single bonds, C=O double bonds and carboxyl groups, respectively [16]. The peak at lower binding energy is assigned to sp^2-like carbon. The presence of this double-bonded carbon is independently

confirmed in our C K-edge X-ray absorption spectra (data not shown). The surface sp^2 carbon is believed to act as acceptor and lead to a surface band bending which is considered relevant in the transformation between NV^0 and NV^- [17]. The spectra with high and low PL background, however, do not differ strongly enough to conclude unambiguously on the reason for the PL background.

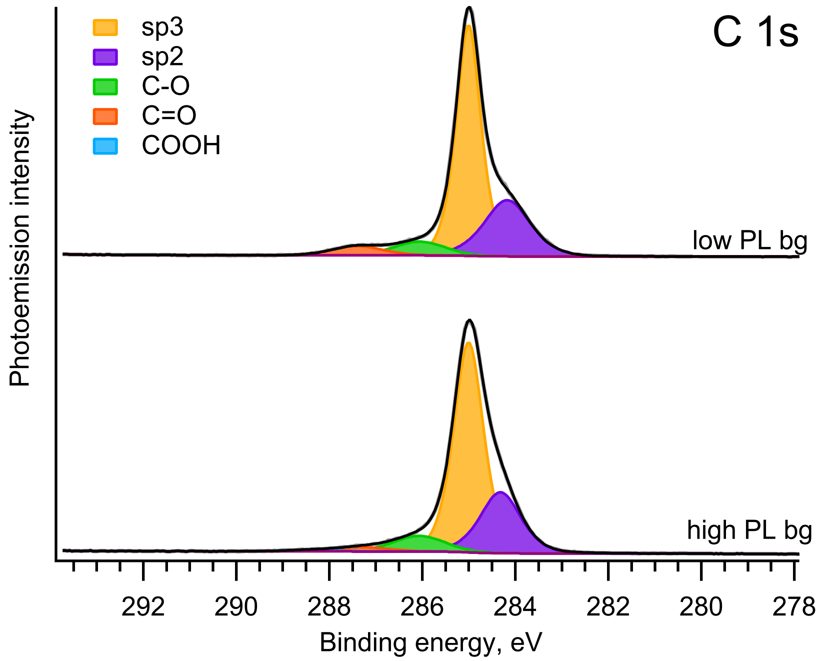

Figure S2: XPS measurements on a reference sample with low PL background (top) and on the sample with high PL background (bottom).

S5 Supplementary figures

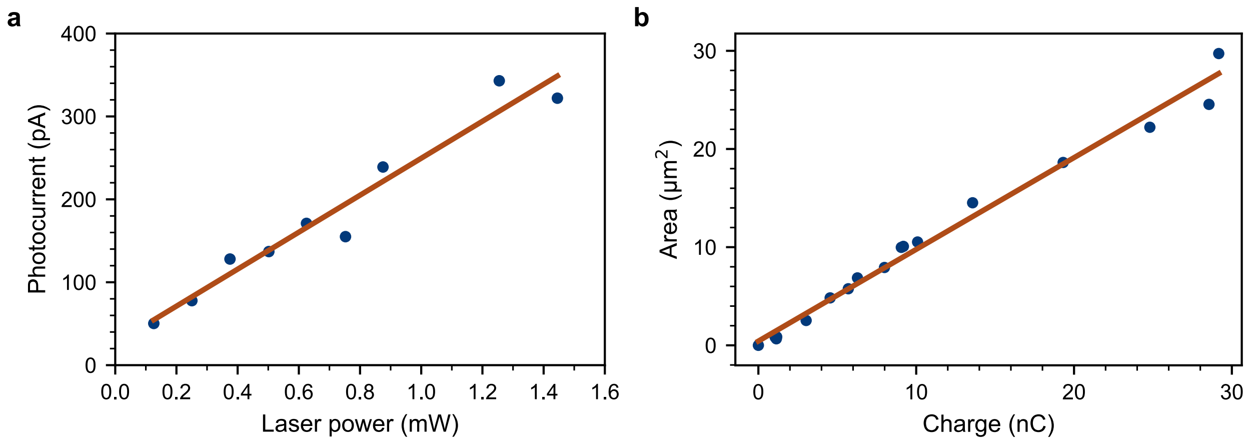

Figure S3: (a) Photocurrent as a function of the laser power measured before the objective at a constant voltage of +10 V. The data (dark blue points) is fitted with a linear function (brown line), which implies a single-photon excitation of charge carriers. (b) Area of the PL-bleached region as a function of the total charge flown through the cantilever during laser scanning experiments at a constant laser power of 500 μW and increasing voltage from 0 V to 20 V. The data (dark blue points) is fitted with a linear function (brown line).

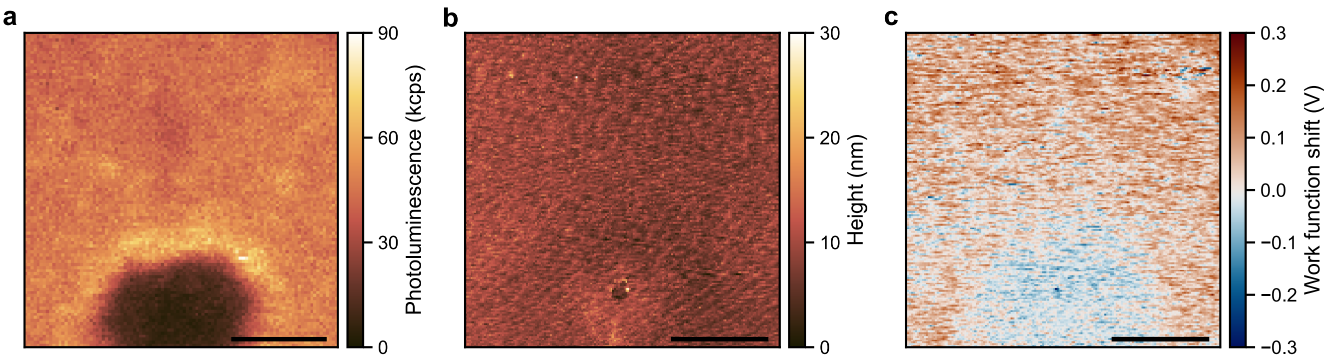

Figure S4: (a) PL map of the sample surface with the reduced background PL. (b) Topography AFM image showing the sample surface in the same area as in a). (c) KPFM image showing the contact potential difference between the sample surface and the cantilever in the same area as in a). The scale bars are 2 μm.

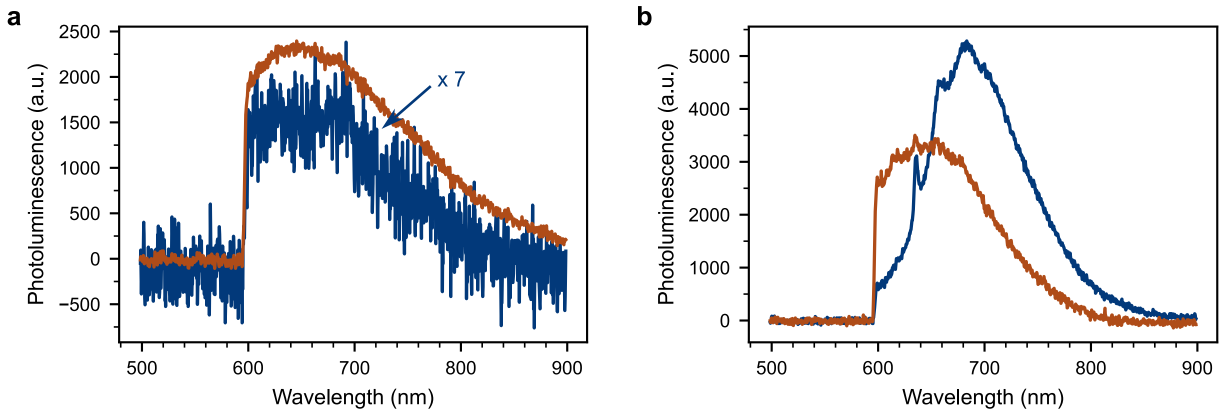

Figure S5: (a) PL spectra from the background before (brown) and after (blue) the application of photocurrent. The spectrum after the c-AFM experiment is magnified by a factor of 7 to allow the shape comparison. (b) PL spectra from the NV center before (brown) and after (blue) the application of photocurrent. To obtain these spectra, the respective background PL data shown in a) were subtracted from the measured spectra. Each spectrum measurement consisted of 3 accumulations with a duration of 20 seconds.

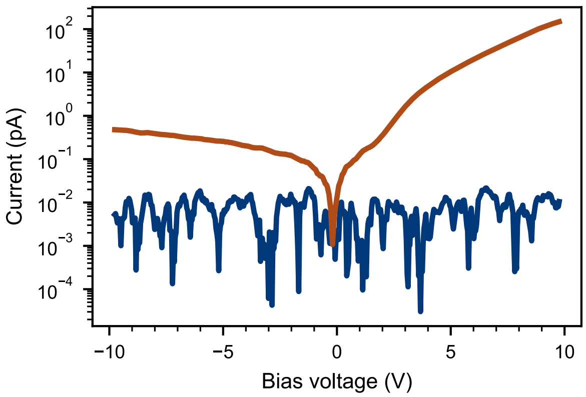

Figure S6: Current-voltage characteristic measured in the dark (blue) and under the laser illumination (brown). The laser with the power of 500 µW was focused on the edge of the surface electrode.

S6 References

[1] J. Fuhrmann, J. Lang, J. Scharpf, N. Striegler, T. Unden, P. Neumann, J. Bansmann, and F. Jelezko, "Probing coherence properties of shallow implanted nv ensembles under different oxygen terminations," *Materials for Quantum Technology*, vol. 4, p. 041001, 2024.

[2] J. Wang, W. Zhang, J. Zhang, J. You, Y. Li, G. Guo, F. Feng, X. Song, L. Lou, W. Zhu, and G. Wang, "Coherence times of precise depth controlled NV centers in diamond," *Nanoscale*, vol. 8, no. 10, pp. 5780–5785, 2016.

[3] M. V. Hauf, B. Grotz, B. Naydenov, M. Dankerl, S. Pezzagna, J. Meijer, F. Jelezko, J. Wrachtrup, M. Stutzmann, F. Reinhard, and J. A. Garrido, "Chemical control of the charge state of nitrogen-vacancy centers in diamond," *Physical Review B*, vol. 83, p. 081304, Feb. 2011.

[4] K.-M. C. Fu, C. Santori, P. E. Barclay, and R. G. Beausoleil, "Conversion of neutral nitrogen-vacancy centers to negatively charged nitrogen-vacancy centers through selective oxidation," *Applied Physics Letters*, vol. 96, p. 121907, Mar. 2010.

[5] S. Trofimov and B. Naydenov, "Combined Confocal-Atomic-Force Microscope Setup for Quantum Sensing Applications with Sub-diffractional Spatial Resolution," *physica status solidi (a)*, p. 2400541, Sept. 2024.

[6] J. M. Binder, A. Stark, N. Tomek, J. Scheuer, F. Frank, K. D. Jahnke, C. Müller, S. Schmitt, M. H. Metsch, T. Unden, T. Gehring, A. Huck, U. L. Andersen, L. J. Rogers, and F. Jelezko, "Qudi: A modular python suite for experiment control and data processing," *SoftwareX*, vol. 6, pp. 85–90, 2017.

[7] S. Fedoseenko, D. Vyalikh, I. Iossifov, R. Follath, S. Gorovikov, R. Püttner, J. Schmidt, S. Molodtsov, V. Adamchuk, W. Gudat, and G. Kaindl, "Commissioning results and performance of the high-resolution Russian-German Beamline at BESSY II," *Nucl. Instrum. Methods Phys. Res. A*, vol. 505, p. 718–728, 2003.

[8] R. Stangl and C. Leendertz, "General Principles of Solar Cell Simulation and Introduction to AFORS-HET," in *Physics and Technology of Amorphous-Crystalline Heterostructure Silicon Solar Cells* (W. G. J. H. M. Van Sark, L. Korte, and F. Roca, eds.), vol. 0, pp. 445–458, Berlin, Heidelberg: Springer Berlin Heidelberg, 2012. Series Title: Engineering Materials.

[9] M. Rieger, V. Villafañe, L. M. Todenhagen, S. Matthies, S. Appel, M. S. Brandt, K. Müller, and J. J. Finley, "Fast optoelectronic charge state conversion of silicon vacancies in diamond," *Science Advances*, vol. 10, p. eadl4265, Feb. 2024.

[10] A. Chemin, I. Levine, M. Rusu, R. Vaujour, P. Knittel, P. Reinke, K. Hinrichs, T. Unold, T. Dittrich, and T. Petit, "Surface-Mediated Charge Transfer of Photogenerated Carriers in Diamond," *Small Methods*, vol. 7, p. 2300423, Nov. 2023.

[11] S. Sze, D. Coleman, and A. Loya, “Current transport in metal-semiconductor-metal (MSM) structures,” *Solid-State Electronics*, vol. 14, pp. 1209–1218, Dec. 1971.

[12] R. Vanselow and X. Li, “The work function of kinked areas on clean, thermally rounded Pt and Rh crystallites: its dependence on the structure of terraces and edges,” *Surface Science*, vol. 264, pp. L200–L206, Mar. 1992.

[13] H. Kiyota, H. Okushi, T. Ando, M. Kamo, and Y. Sato, “Electrical properties of a Schottky barrier formed on a homoepitaxially grown diamond (001) film,” *Diamond and Related Materials*, vol. 5, pp. 718–722, May 1996.

[14] L. A. Völker, K. Herb, D. A. Merchant, L. Bechelli, C. L. Degen, and J. M. Abendroth, “Charge and spin dynamics and destabilization of shallow nitrogen–vacancy centers under uv and blue excitation,” *Nano Letters*, vol. 24, pp. 11895–11903, 2024.

[15] C. Pederson, N. S. Yama, L. Beale, M. Markham, M. E. Turiansky, and K.-M. C. Fu, “Rapid, in situ neutralization of nitrogen- and silicon-vacancy centers in diamond using above-band gap optical excitation,” *Nano Letters*, vol. 25, no. 2, pp. 673–680, 2025.

[16] S. Sangtawesin, B. L. Dwyer, S. Srinivasan, J. J. Allred, L. V. H. Rodgers, K. D. Greve, A. Stacey, N. Dontschuk, K. M. O’Donnell, D. Hu, D. A. Evans, C. Jaye, D. A. Fischer, M. L. Markham, D. J. Twitchen, H. Park, M. D. Lukin, and N. P. de Leon, “Origins of diamond surface noise probed by correlating single-spin measurements with surface spectroscopy,” *Phys. Rev. X*, vol. 9, p. 031052, 2019.

[17] A. Stacey, N. Dontschuk, J.-P. Chou, D. A. Broadway, A. K. Schenk, M. J. Sear, J.-P. Tetienne, A. Hoffman, S. Prawer, C. I. Pakes, A. Tadich, N. P. de Leon, A. Gali, and L. C. L. Hollenberg, “Evidence for primal sp2 defects at the diamond surface: Candidates for electron trapping and noise sources,” *Advanced Materials Interfaces*, vol. 6, no. 3, p. 1801449, 2019.
